# Cyriax syndrome in a young male professional soccer player: A case report

**DOI:** 10.17159/2078-516X/2020/v32i1a8300

**Published:** 2020-01-01

**Authors:** CT Panka, PY Tchebegna

**Affiliations:** 1 General Surgery, Sport and Exercise Medicine, Health and Sports Clinic, Douala, Cameroon; 2 Kerthan Clinic, Douala, Cameroon

**Keywords:** chest pain, ribs, lidocaine infiltrations

## Abstract

Cyriax syndrome is a neglected cause of lower chest or upper abdominal pain. The pain is due to an irritation of the intercostal nerves by the incomplete dislocation of the costal cartilage of the ribs. This study presents a rare case of a young male soccer player with cyriax syndrome and offers medical insight into the clinical diagnosis and one therapeutic modality of this condition. He came to the sports clinic reporting severe pain in the right lower rib cage near the costal cartilage junction which he has experienced for about three weeks. A chest x-ray detected nothing. After the failure of initial conservative measures, persistence of the pain led authors to initiate two separate local infiltrations using 1% lidocaine ten days apart. This resulted in a gradual decrease in pain. Cyriax syndrome is an unrecognised pathology, thus it is important for team physicians to consider this diagnosis when assessing athletes with persistent chest pain.

Cyriax syndrome is characterised by pain due to the irritation of the intercostal nerve as a result of the incomplete dislocation of the costal cartilage of the 8th, 9th or 10th ribs. It is a neglected cause of lower chest or upper abdominal pain with an unpredictable development. This study presents a rare case of a young male soccer player with slipping rib syndrome and offers medical insight to the clinical diagnosis and therapeutic modality of this condition.

## Case report

A 24-year-old male professional soccer player visited the sports clinic for right hypochondrium pain which had been developing for about three weeks prior to consultation. During a football match in which the player was competing, after jumping up in the air, he received a direct shock to his chest from his opponent’s knee. He then felt a severe, throbbing and shooting pain in the area of his left lower rib cage (intensity 8/10 according to the visual analog scale for pain) aggravated by dorsal and lateral decubitus but felt better when he was in a standing or seated position. Two days later he decided to consult the team physiotherapist who carried out conservative treatment for two weeks without any improvement.

**Fig. 1:**
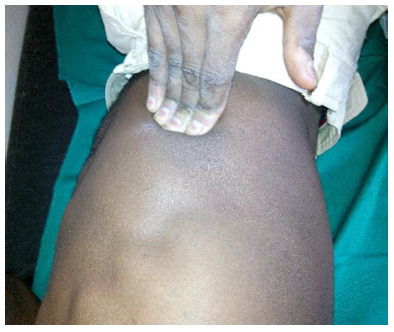
Pressure exerted on the ribs to reproduce the pain

During the initial injury evaluation, the player reported that he could not sleep well due to the severe pain in the right lower rib cage near the costal-cartilage junction. The pain was replicated with rib pressure (Fig. 1), with elective pain on palpation of the costal rim of the 9th and 10th ribs, during the Valsalva manoeuvre and with sit-up tests. No deformity or skin changes were observed.

The player had normal hemodynamic parameters of blood pressure: 110/60 mm hg, and his pulse rate was 58 beats/min. The hooking manoeuvre (curling the fingers beneath the costal rim and then instigating an upward lift) was positive without any nerve block. Traction on the ribs resulted in pain when they were pushed out.

A diagnostic chest x-ray was performed which showed no signs of rib fracture.

**Fig. 2:**
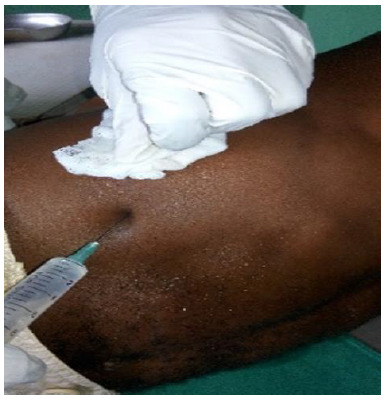
Local infiltrations with 1% lidocaine

Conservative therapy was begun with oral analgesics (Tramadol 50mg BID, Acetaminophen 1G TID) and nonsteroidal anti-inflammatory drugs (Diclofenac 50 mg BID) for one week. During this period, the patient was encouraged to not participate in sporting activities. Dissatisfied with the persistent pain he was experiencing, he returned two weeks later complaining of the same symptoms. The persistence of this right basithoracic pain resulted in the authors establishing two separated series of local infiltrations with 1% lidocaine 10 days apart (Fig. 2). Thereafter, light rehabilitation exercises were initiated over a period of one month. A gradual decrease in pain was observed and the player was able to resume his professional activity after three months.

This case is unique in that this player was diagnosed with a serious chest condition seldom mentioned in current sports medicine practice manuals. Thus, this condition has the potential to be misdiagnosed by team doctors. The participant was informed about all aspects of the study and gave his informed and signed consent for publication.

## Discussion

Slipping rib syndrome was first described in 1919 by Cyriax.,^[1]^ an English orthopaedic surgeon. The cause is most often traumatic, either directly or indirectly, which often goes unnoticed. Spontaneous evolution is unpredictable and often paroxysmal. However, many healthcare professionals involved with diagnosing and treating athletes and other active individuals are often unfamiliar with this musculoskeletal condition.^[2]^

Indeed, the dislocated chondral end compresses the intercostal nerve which runs under the upper rib. The pain then spreads down to the right or left hypochondrium in the sensitive skin area of the intercostal nerve. There are very few publications on the prevalence of this syndrome. Monnin et al.’s study has shown it to be 1% ^[3]^ and 1.5% in the study by Sanou et al. in Gabon. ^[6]^ In almost all cases, women are more affected. The low prevalence of this condition may be due to misdiagnosis and underdiagnosis, as it is very rare.^[3]^

In most studies, from the clinical perspective, the pain is abdomino-thoracic, triggered or worsened by certain movements, developing over several days or months, more often touching the 10th rib in negative paraclinical examinations. ^[4–6]^ The findings in other studies were similar to those highlighted in this case. This patient had a right hypochondralgia aggravated by the lateral and dorsal decubitus which developed over three weeks. He sought the care of a physiotherapist without experiencing any improvement. Most of the time, the trauma occurs during a high level football match, but it can also occur in other sports, such as golf, tennis, rugby or boxing.^[4,7]^ It is necessary to know how to differentiate this pathology from the Tietze syndrome (painful inflammation of the upper chondro-costal and sterno-clavicular joints) and abdominal infections (gastroesophageal reflux, functional colopathy, vesicular lithiasis).^[4]^

Treatment is initially conservative, where analgesics are used, and if the pain persists, local infiltration can be undertaken. ^[4,8]^ In this study, two infiltrations were done (within the space of one week) and this patient was relieved of the pain. If the conservative approach fails, surgical treatment, consisting of resection of the last five to eight cm of dislocated cartilage is considered. ^[4,6]^

Cyriax syndrome is an unrecognised pathology often confused with several painful abdominal and thoracic pathologies. Its diagnosis is based on the existence of elective pain at the pressure of the costal edge of the ribs with negative radiological examinations. It is important for team physicians to consider the diagnosis of Cyriax syndrome when assessing athletes with persistent thoracic or abdominal pain.
